# Population genomics provides insights into the genetic diversity and adaptation of the *Pieris rapae* in China

**DOI:** 10.1371/journal.pone.0294521

**Published:** 2023-11-16

**Authors:** Linlin Zheng, Huan Wang, Junjie Lin, Yuxun Zhou, Junhua Xiao, Kai Li

**Affiliations:** 1 College of Biological Science and Medical Engineering, Donghua University, Songjiang District, Shanghai, China; 2 Department of Plant Science and Technology, Shanghai Vocational College of Agriculture and Forestry, Shanghai, China; Huazhong University of Science and Technology, CHINA

## Abstract

The cabbage white butterfly (*Pieris rapae*), a major agricultural pest, has become one of the most abundant and destructive butterflies in the world. It is widely distributed in a large variety of climates and terrains of China due to its strong adaptability. To gain insight into the population genetic characteristics of *P*. *rapae* in China, we resequenced the genome of 51 individuals from 19 areas throughout China. Using population genomics approaches, a dense variant map of *P*. *rapae* was observed, indicating a high level of polymorphism that could result in adaptation to a changing environment. The feature of the genetic structure suggested considerable genetic admixture in different geographical groups. Additionally, our analyses suggest that physical barriers may have played a more important role than geographic distance in driving genetic differentiation. Population history showed the effective population size of *P*. *rapae* was greatly affected by global temperature changes, with mild periods (i.e., temperatures warmer than those during glaciation but not excessively hot) leading to an increase in population size. Furthermore, by comparing populations from south and north China, we have identified selected genes related to sensing temperature, growth, neuromodulation and immune response, which may reveal the genetic basis of adaptation to different environments. Our study is the first to illustrate the genetic signatures of *P*. *rapae* in China at the population genomic level, providing fundamental knowledge of the genetic diversity and adaptation of *P*. *rapae*.

## Introduction

Exploring the effects of changeable environments on genetic diversity of a species can elucidate its evolutionary history and underlying adaptations [1]. Pest populations have garnered increasing attention for their remarkable ability to rapidly evolve and adapt to changing environments [2]. The capacity of pests to evolve resistance rapidly to insecticides and host-plant resistance presents a continuous challenge for pest management [3]. Given that Lepidopteran species are amongst the most destructive pests of food and fiber crops worldwide, the need for control tactics that are not only effective but also more environmentally friendly is becoming increasingly urgent [4]. A comprehensive understanding of the genetic divergence and adaptive traits among different pest populations is a prerequisite for devising effective pest control strategies [5, 6].

The notorious cabbage white butterfly, *Pieris rapae*, is a lepidopteran pest whose larvae feed on plenty of cash crops such as *Brassica juncea*, *Cleome spinosa* and *Tropaeolum majus* [7]. Its larval droppings also cause soft rot, seriously impacting the quality of crops. With the expansion of human trade routes and the diversification of brassicaceous crops, *P*. *rapae* has been dispersed from eastern Europe to inhabit every continent except South America and Antarctica [8, 9]. In mainland China, the natural range of the cabbage white butterfly encompasses diverse environments, from tropical to temperate climates zones and from plains to mountainous areas. Despite the presence of numerous natural enemies and pesticides [10, 11], that can impede the growth of *P*. *rapae*, this butterfly still causes considerable economic losses in China annually. *P*. *rapae* has adapted to an abundant and predictable agricultural resource through a narrowing of niche breadth and loss of genetic variants [12]. Exploring the population genetic diversity of *P*. *rapae* is not only essential for understanding the distribution pattern of this butterfly, but also has great implications for uncovering the genetic basis of adaptation to the local environment.

At present, studies on *P*. *rapae* have mainly focused on morphology, control methods, immune mechanisms and transcriptomics [13–16]. However, knowledge of the evolution and adaptation of *P*. *rapae* is limited due to the lack of studies at the genomic level. Population genomics of *P*. *rapae* has been hampered by limitations in obtaining sufficient samples and high-resolution molecular markers. Spurred by rapid developments in sequencing technology, increased data production is accompanied by a drive to develop efficient computational approaches to interpret patterns of genetic variation at the genomic scale [17]. Genome-wide population surveys of natural populations promise insights into the evolutionary processes and the genetic basis underlying speciation [18]. In recent years, the draft genome of *P*. *rapae* has been sequenced and annotated successively [19], providing a foundation for further population genomic studies.

In this study, 19 feral colonies of *P*. *rapae* from different locations across China were collected and the whole genomes of a total of 51 individuals were resequenced. We investigated genomic polymorphisms, population structure, and demographic history in these butterfly populations. Moreover, the rapidly evolving genes related to adaptation to local environments in *P*. *rapae* were also identified. This study provides insights into the genetic diversity and evolutionary history of *P*. *rapae*, as well as theoretical support for the population genome study of the cabbage white butterfly.

## Materials and methods

### Sampling and sequencing

51 adult butterflies were collected from 19 areas in China, covering multiple climates from temperate to tropical and various terrains such as plains, mountains, basins, and islands. These butterflies have been preliminarily divided into four major populations based on their geographical location, with 18 individuals in the north population, 10 in the coastal population, 11 in the southeast population and 12 in the southwest population (Specific grouping information is provided in S1 Table). For each individual, the 350-bp paired-end libraries with high-quality DNA were prepared by TruSeq Library Construction Kit (illumina®, San Diego, CA, USA) (S2 Table). We sequenced the DNA on the Illumina HiSeqTMPE150 (illumina®, San Diego, CA, USA) sequencing platform using standard procedures.

### Quality control and reads mapping

To ensure the accuracy of the raw sequencing data, it is essential to eliminate any interfering data, including adapters, low-quality bases, and undetected bases (expressed in N). We employed fastp [20] to filter the raw sequencing reads to obtain high-quality clean data. The adapter in the reads was automatically detected and removed. Paired reads were discarded if the number of N’s in any of the paired reads exceeded 10%. Additionally, the number of low quality (Q ≤ 5) bases in a single read was restricted to less than 50%. Then our in-house python script was utilized to assess the filtered high-quality data.

95.8 Gb of clean reads were retained and mapped to the latest version of the *P*. *rapae* reference genome (https://ftp.ncbi.nlm.nih.gov/genomes/all/GCF/905/147/795/GCF_905147795.1_ilPieRapa1.1) using Burrows-Wheeler Alignment (BWA) aligner [21] with the option “-t 18 -k 32 -M”. Alignment bam files were sorted by SAMtools [22]. Polymerase chain reaction (PCR) duplicates were removed using sentieon [23] with the following set of parameters: driver -t 30—algo Dedup–rmdup. Finally, Flagstat from Samtools was used to evaluate the mapping rate. Qualimap’s bamqc [24] was used to calculate depth and coverage.

### Variants calling and genotyping

Single nucleotide polymorphisms (SNPs) and insertion-deletion sites (INDELs) were detected using sentieon [23] with the relevant parameters: sentieon driver -t 30—algo Haplotyper—emit_mode GVCF. About 2.3 × 10^7^ raw SNPs were detected. To obtain reliable SNPs, we set the following filtering criteria using vcfutils and vcftools [25]: (1) SNPs within 5 bp around an indel were filtered. (2) At least 5 bp between two SNPs. (3) The minimum allele frequency (MAF) of SNPs should be ≥ 0.05. (4) SNPs with a missing rate ≥ 30% were removed. (5) The sum of read depths across all populations was [4, 2000]. (6) Only SNPs with a quality score greater than 20 are retained.

After applying the above filters, high-quality clean data were retained for downstream analyses. Subsequently, the table_annovar.pl script provided by the ANNOVAR [26] was used to annotate SNPs and INDELs respectively. To provide a clear picture of variant characteristics, the variation type and density in different chromosomes were calculated by a custom script written in Perl.

In addition, we utilized BreakDancer [27] to detect structural variation (SVs) in each sample, removing SV sites with a quality score of less than 40 and support read counts of less than 10. CNVnator [28] was used to detect copy number variations (CNVs) with the option “-his 300”. Raw CNVs with a length ≤ 1000 bp were removed. Furthermore, a CNV was discarded if the multiple aligned reads of the CNV were greater than 50%.

### Population structure

To further estimate individual admixture, we used ADMIXTURE v1.23 software [29] to investigate potential population structure, with presumed ancestral clusters (K) ranging from 2 to 5. A modified SNP dataset with linkage disequilibrium (LD) correlation coefficient (r^2^) ≤ 0.2 was used as the input file. The optimal K was determined by the lowest cross-validation error. Principal component analysis (PCA) with biallelic SNPs was performed using the PLINK tool [30], and the first four significant components were plotted. To infer the phylogenetic relationship between different geographical groups, a phylogenetic tree based on the maximum likelihood method was constructed using iqtree2 software [31] with 1000 bootstraps. ITOL online website (https://itol.embl.de/) was then utilized to visualize the phylogenetic tree.

### Demographic history

We applied the pairwise sequentially Markovian coalescent (PSMC) model analysis [32] to trace potential historical fluctuations of effective population size. The short sequence on the scaffold was removed, and only the sequence on the chromosome was retained for subsequent analysis, with parameters set as -N 25 -t 15 -r 5 -p 4+25*2+4+6. A psmc_plot.pl script (provided with the PSMC software) was used for plotting. The mutation rate was set to 2.5 × 10^−9^ per site per generation [19], and the average generation time (g) was set to 4 years. TreeMix [33] was applied to detect gene flow, using an SNPs dataset with LD correlation coefficient (r^2^) ≤ 0.2 as the input file. A maximum likelihood tree was inferred according to the hypothetical number of gene flows, and the direction of gene flow was calibrated on the tree.

### Population genetic differentiation

Genetic differentiation index (F_ST_) between populations, nucleotide diversity (θπ), and Tajima’s D was calculated using vcftools software [25], with a window size (-fst-window-size) of 100 Kb and a step size (—fst-window-step) of 10 Kb. PLINK [30] was used to calculate the heterozygosity of the four populations separately, and a custom R script was used to plot the results.

### Linkage Disequilibrium decay

To assess the Linkage Disequilibrium (LD) pattern in cabbage white butterflies, PopLDdecay software [34] was used to calculate the correlation coefficient (r^2^) between any two loci in each population, with the parameter of MaxDist set to 2, i.e. the maximum distance calculated by r^2^ was 2 Kb. The LD decay curve was then plotted using a Plot_MultiPop.pl script (provided with the PopLDdecay software).

### Genomic signatures of selection

To further investigate the effects of different latitudes or climates on this butterfly, we combined the *P*. *rapae* individuals from southwestern and southeastern areas into south populations to compare with north populations. In general, positive selection results in higher genetic differentiation of loci between populations and lower genetic diversity within populations [35]. In order to detect selection signals associated with local adaptation, we used vcftools to calculate the genome-wide distribution of F_ST_ values and θπ ratios with a sliding-window method (100-kb windows with 10-kb step). Z transformation for F_ST_ values and log_2_ transformation for θπ ratios were applied. The windows with the top 5% Z(F_ST_) and log_2_ (θπ ratio) values were simultaneously considered as the candidate outliers under strong selection, and the genes in the outlier windows were screened as selected genes.

In an effort to further explore the role of these selected genes in the adaptive evolution of *P*. *rapae*, we performed Gene Ontology (GO) and Kyoto Encyclopedia of Genes and Genomes (KEGG) pathway analysis. The reference coding sequence of *P*. *rapae* was proactively annotated on the eggNOG-MAPPER online website (http://eggnog-mapper.embl.de/) to obtain GO and KEGG background annotation information. An R package, clusterProfiler [36], was utilized to automate the enrichment of selected genes located in specific regions. The hypergeometric test was applied to estimate significance (P < 0.05, q < 0.05).

## Results

### Genomic variations

**Fig 1:**
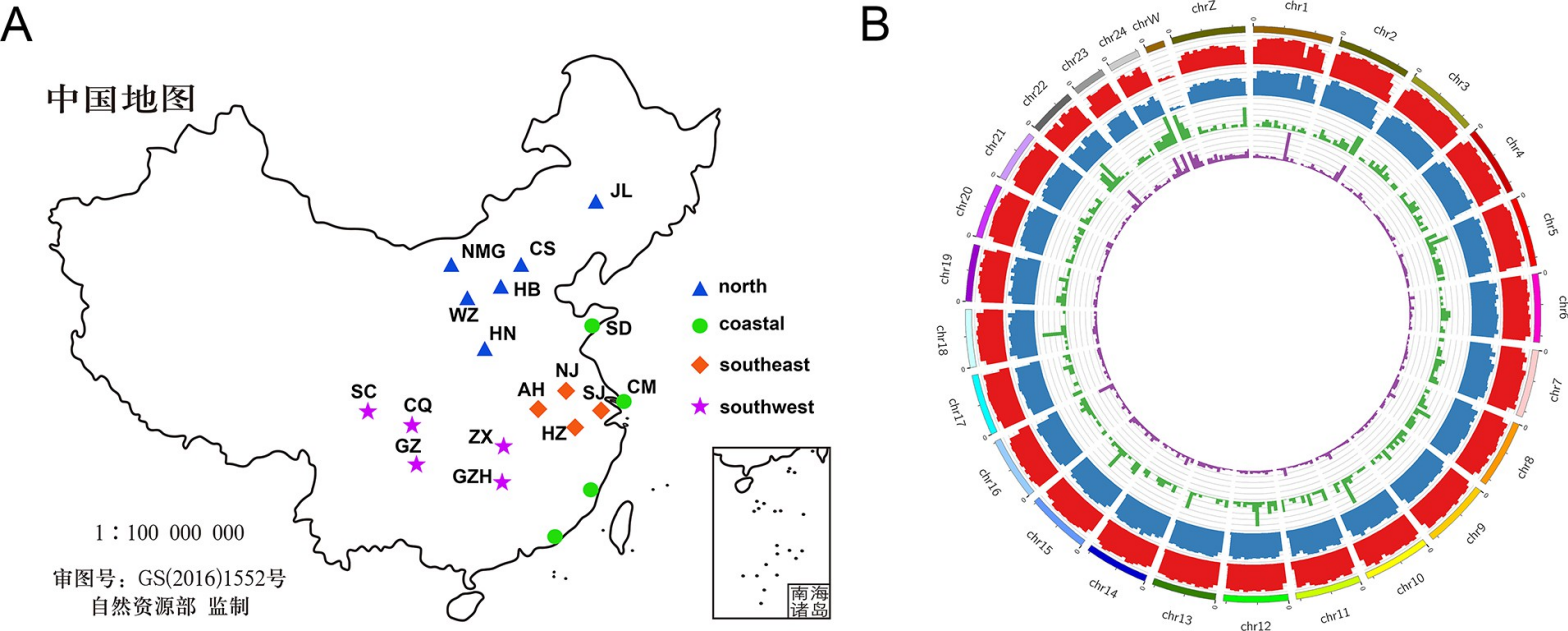
Sampling and variation. (A) The geographical location of *P*. *rapae* sampling in 19 areas of China, with an average of 3 individuals per location. (The ground map was reprinted from Standard Map Service (http://bzdt.ch.mnr.gov.cn) under a CC BY license, with permission from the Ministry of Natural Resources of the People’s Republic of China under the permission number GS(2019)1552, original copyright 2019; the ground map is available at: http://bzdt.ch.mnr.gov.cn/browse.html?picId=%224028b0625501ad13015501ad2bfc0002%22). (B) The density distribution of different variants on the chromosomes of *P*. *rapae* (from the outermost to the innermost circles represent chromosomes, SNPs, INDELs, SVs, and CNVs density maps, respectively).

We performed whole-genome resequencing for 51 cabbage white butterflies (Fig 1A), with a total of 115 Gb of raw sequencing data. After stringent quality filtering, high quality cleaning data was obtained with an effective base of 99.84% and an average Q20 of 98.27% for further analysis (S3 Table). Genome alignment of *P*. *rapae* indicated an average depth of 9.5× and coverage of 93% relative to the reference genome (S4 Table). A total of 4,696,801 reliable SNPs and 894,367 reliable INDELs were identified between different populations after quality control, suggesting an average of 19 SNPs and 3.6 INDELs per Kb of the genome. In addition, we determined 3,941 CNVs and 974 SVs, all of which contributed to a comprehensive genetic variation map for *P*. *rapae* (Fig 1B). Obviously, a very dense variant map was generated for this butterfly, revealing a high level of polymorphism.

### Characteristics of SNPs and INDELs

**Fig 2:**
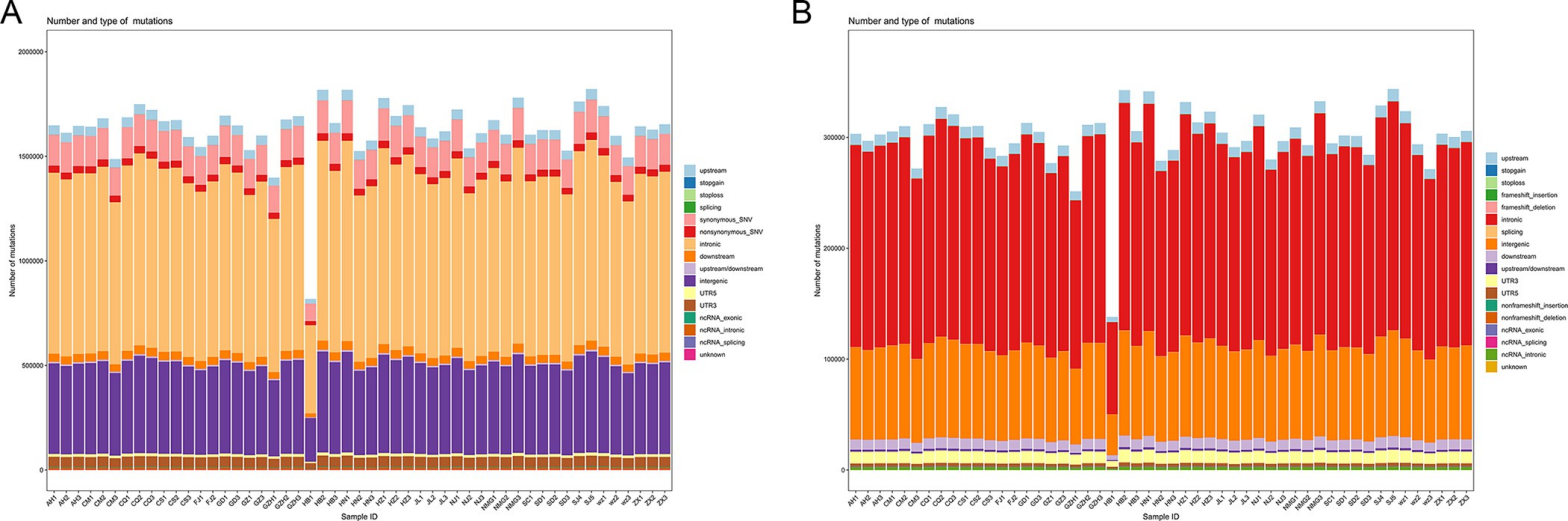
ANNOVAR annotation results. (A) SNP annotation results for each *P*. *rapae* individual. (B) INDELs annotation results for each *P*. *rapae* individual.

**Fig 3:**
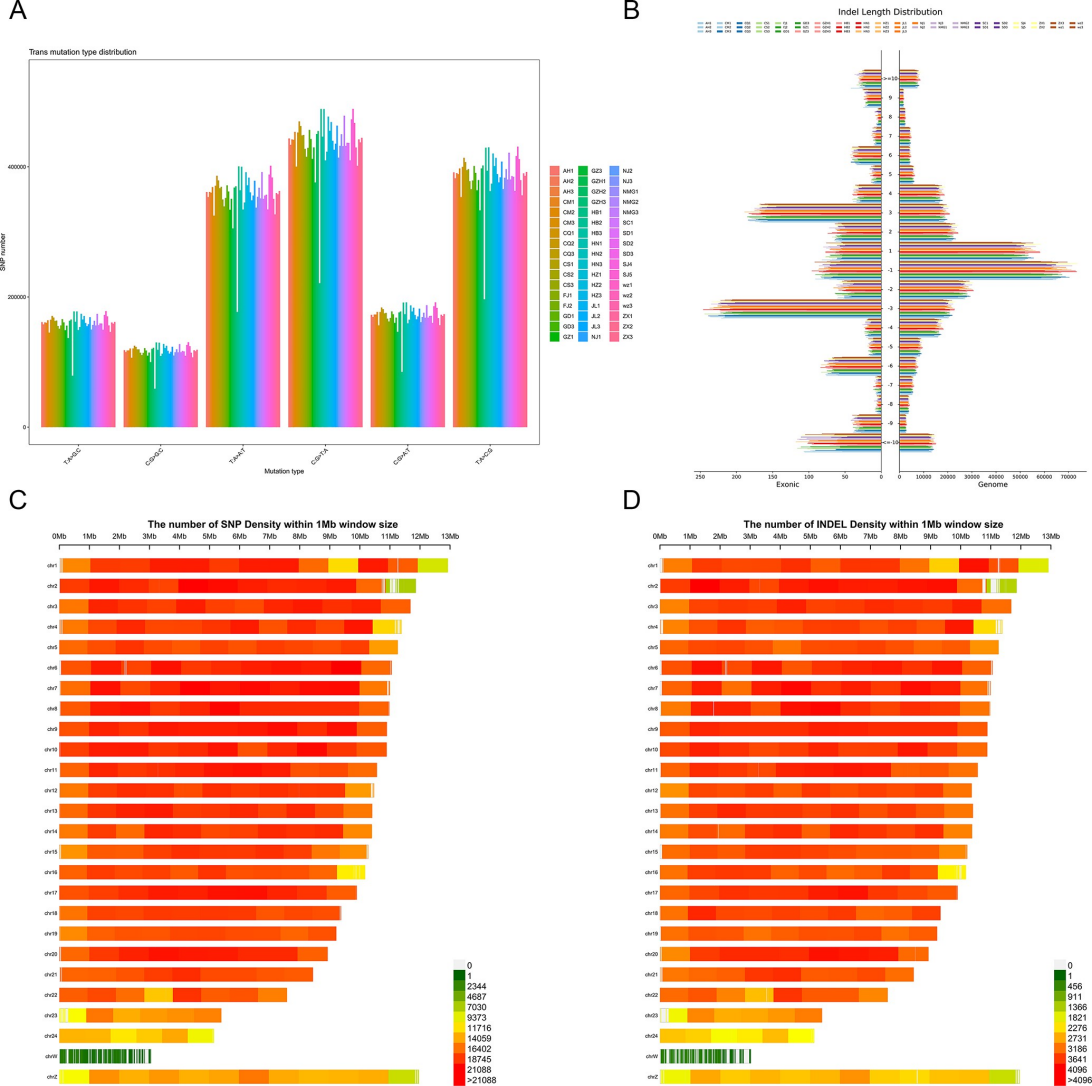
Variation characteristics. (A) SNPs mutation base in different individuals of *P*. *rapae*. (B) Length distribution of INDELs in exons and genome. (A positive number indicates an insert, and a negative number indicates a deletion). (C) SNPs density distribution on chromosomes (1Mb as a window). (D) INDELs density distribution on chromosomes (1Mb as a window).

Through our annotation analysis, we have observed 459,845 SNPs in the coding region, accounting for 9.7% of all SNPs (Fig 2A). Among these SNPs, 365,873 were synonymous mutations, while 92,680 were non-synonymous mutations. Additionally, we found 2,841 INDELs in the exonic region, accounting for 0.3% of all INDELs, of which 1016 would result in frameshifts (Fig 2B). Notably, both INDELs and SNPs were mainly distributed in introns and intergenic regions across the genome. The base mutation of SNPs generally tended to be cytosine to thymine and guanine to adenine (C: G > T: A), accounting for almost 30% (Fig 3A). The length of INDELs in exons is often 3 bp or 6 bp, which accounted for 49.2%, as these lengths do not cause a frameshift (Fig 3B). Interestingly, we also observed a significantly reduced density of variants (SNPs and INDELs) on the sex chromosome (Z and W chromosomes) compared to the autosomes (Fig 3C and 3D). This indicates that variation on the sex chromosome is more conserved.

### Population structure

**Fig 4:**
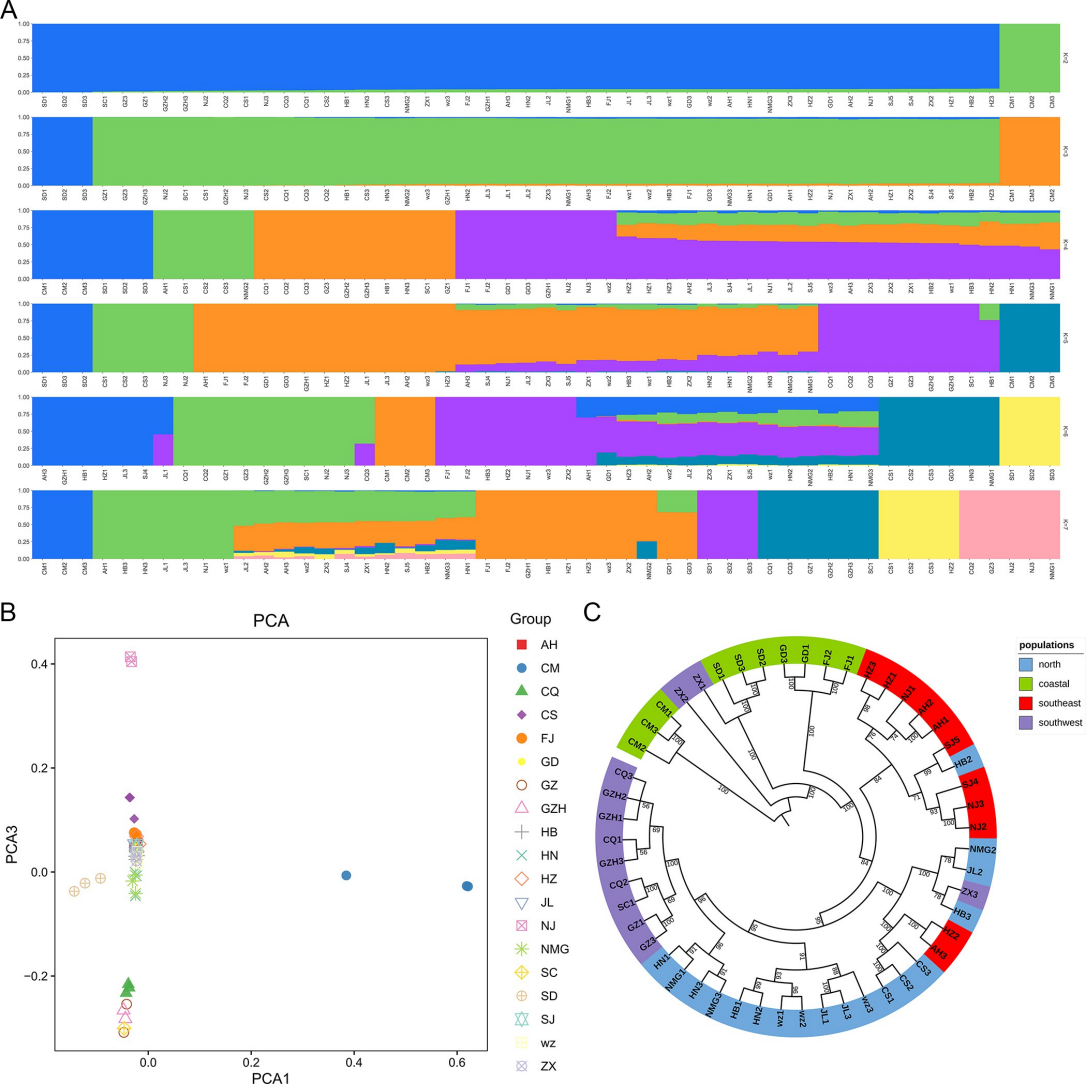
Population structure. (A) Genetic structure of 19 geographical groups. Groupings of samples from two to seven ancestral clusters are shown. (B) Scatter plot of principal components 1 versus 2 (PC1 vs. PC2) for the 19 geographical groups. (C) Phylogenetic rootless trees plotted by maximum likelihood methods (numbers on clades represent bootstrap values).

In order to characterize the relationship among different populations, genetic clustering analysis was performed with varying the number of the presumed ancestor (K). The cross-entropy error rates were lowest for K-values of 2–4 (S1 Fig). When K was 2, individuals from Chongming Island (CM) were distinguished from other populations. With a K value of 3, in addition to CM, Shandong Peninsula (SD) individuals also presented a distinct ancestor. As K increased from 4 to 7, it was observed that the southwest populations (CQ, GZ, SC, GZH) formed an ancestral cluster, while most of the north (JL, WZ, NMG, HB, HN) populations showed different degrees of mixed ancestry (Fig 4A). Principal component analysis (PCA) results further supported these patterns. The first and second principal components (PC1 and PC2) separated CM and SD (S2 Fig), consistent with the ADMIXTURE results at K = 2 and 3; PC1 and PC3 further separated the southwest populations from other populations but were not able to distinguish between north and southeast (Fig 4B and S3 Fig), which was consistent with the ADMIXTURE results at K = 4–7.

Next, a maximum likelihood tree was constructed to infer the phylogenetic relationship between different geographical groups (Fig 4C). We found that geographically close populations were more likely to cluster into a subclade, while some individuals in the southeast and north populations had mixed distributions, which was also consistent with the previous results of ADMIXTURE and PCA. The grouping of populations reflected their geographic locations from the coast to the southwest.

### Demographic history and gene flow

**Fig 5:**
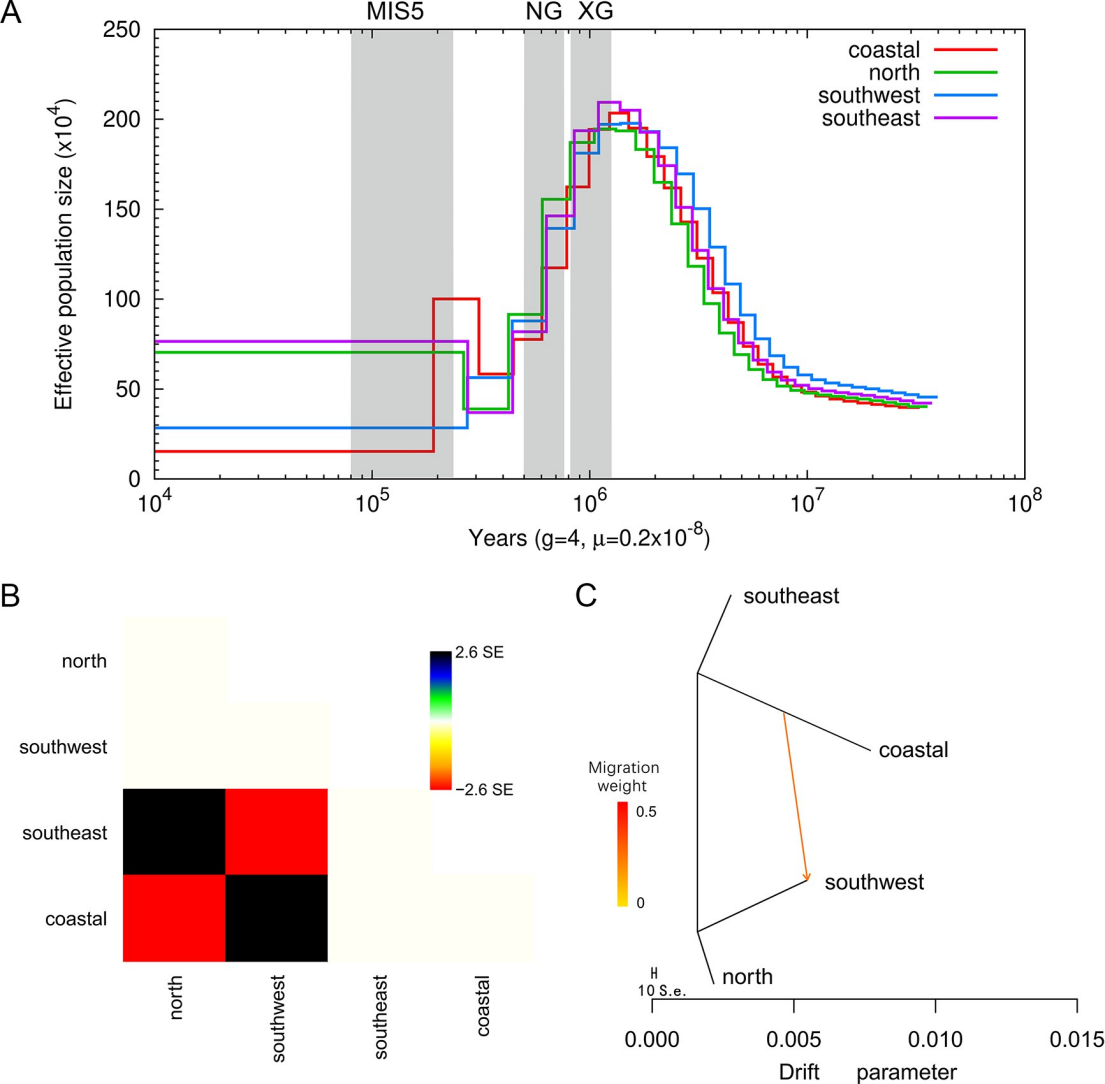
Population history. (A) Historical effective population size analysis of different populations of the butterfly. The period of the Xixiabangma Glaciation (XG, 1,170–800 thousand years ago, kya), Naynayxungla Glaciation (NG, 780–500 kya), and the Marine Isotope Stage 5 (MIS5, 80–130 kya) are shaded in grey. (B) The residual fit is derived from the maximum likelihood tree estimated by TreeMix. Residuals greater than zero indicate that certain populations in the data are more closely related to each other than expected based on the best-fit tree, suggesting the possibility of admixture events. (C) Gene flow (arrow direction indicates the direction of gene flow, arrow color indicates migration weight).

We inferred the historical effective population size to gain a better understanding of the demographic history in this butterfly (Fig 5A and S4 Fig). The population history of the small cabbage white butterfly could be traced back to about 30 million years (Ma) ago. All populations initially experienced similar demographic trajectories: a large population expansion between 1 and 2 million years ago. The population reached a peak approximately 1.5 Ma ago, about 2 × 10^6^. Then, a dramatic population decline of the four populations occurred during Xixiabangma Glaciation (1,170 to 800 thousand years ago) and Naynayyxungla Glaciation (780 to 500 thousand years ago) [37]. Subsequently, a slight population expansion was observed in the north, southeast and coastal populations, roughly coinciding with the Marine Isotope Stage 5 (MIS5, about 130 to 80 thousand years ago) [38], the last major interglacial period of mild temperature in history. The increase in effective population size during the warm periods suggests that an elevated global temperature may be beneficial for *P*. *rapae*. Unlike other populations, the southwest population continued to decline even in warmer climates. Notably, over the last 10 000 years, the effective population sizes of the north and southeast populations have reached nearly 700 000 and 750 000, respectively, which is 3–4 times that of the southwest and coastal populations.

Populations of a species often have complex histories that contribute to their current genetic makeup. For further insight into population history, we also investigated gene flow between populations. Significant gene flow was detected from the coastal population to the southwest population (Fig 5B and 5C). During the global invasion of *P*. *rapae*, the development of the Silk Road of maritime trade likely facilitated the introduction of this butterfly into Asia [9]. It is speculated that coastal populations may have been the historical dispersal source of *P*. *rapae* in China.

### Genetic differentiation, genetic diversity, and LD decay

We calculated F_ST_ to quantify population genetic differentiation (S6 Table). Pairwise F_ST_ values ranged from 0.059 to 0.098, with a mean of 0.074, suggesting only moderate differentiation between populations. Areas with closed terrains, such as the southwest and coastal populations, exhibited a higher F_ST_ of 0.098 compared to other populations.

**Fig 6:**
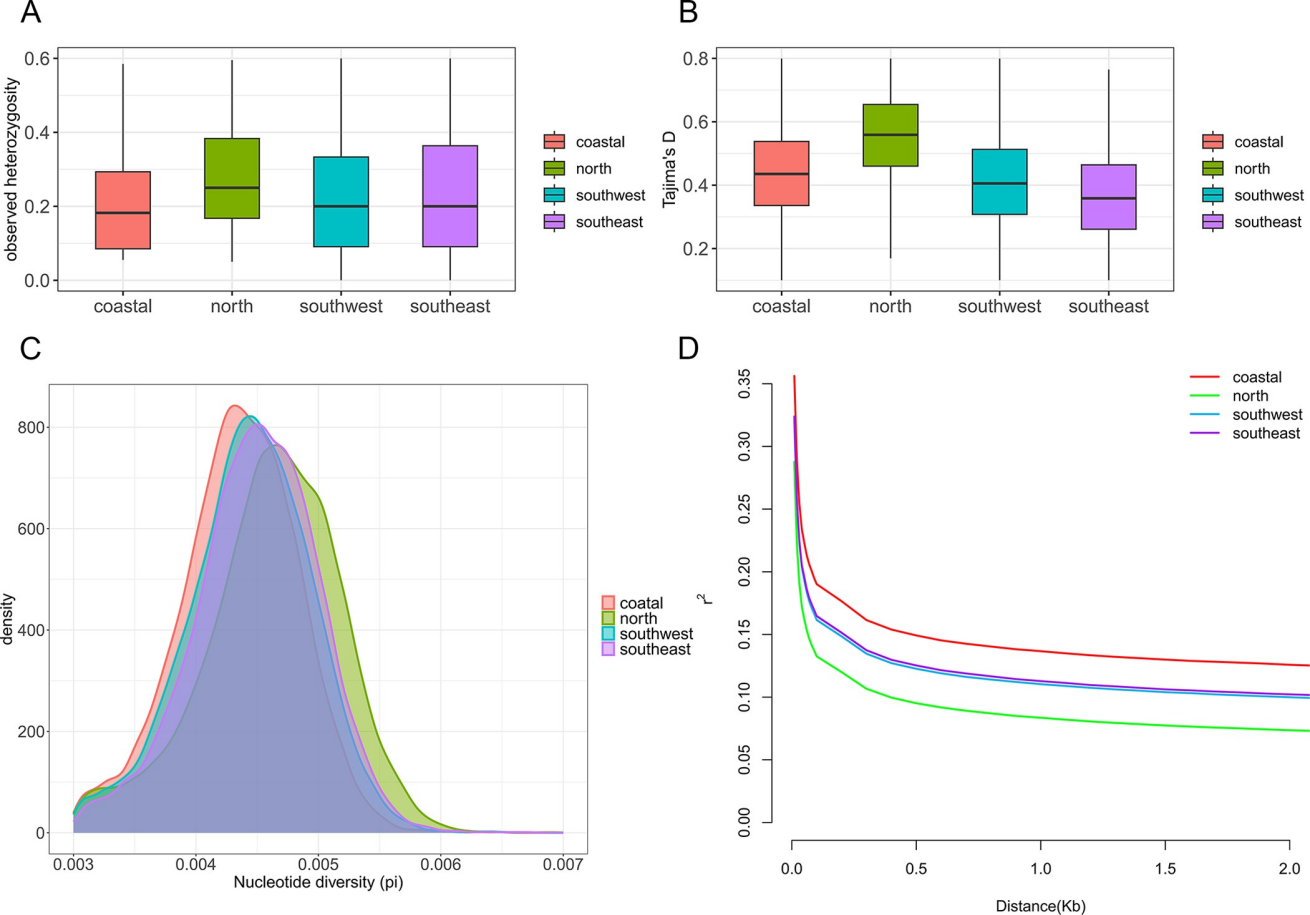
Population genetic differentiation. (A) Observed heterozygosity of the four geographical groups. (B) Tajima’s D values of the four geographical groups. (C) The density of Nucleotide diversity (pi) of the four geographical groups. (D) Linkage-disequilibrium patterns measured with the squared coefficient of correlation (r^2^) of alleles at any two loci using PopLDdecay based on the *P*. *rapae* genome-wide SNPs from different clusters. (north: 62bp, r^2^ = 0.1503; southwest: 139bp, r^2^ = 0.1500; southeast: 148bp, r^2^ = 0.1507; coastal: 441bp, r^2^ = 0.1507).

To explore the genetic diversity among *P*. *rapae* populations, we also calculated the observed heterozygosity, Tajima’s D value and nucleotide diversity (π) using whole genome-wide SNP data. The heterozygosity of the four geographical groups did not differ much, but the level of heterozygosity was high, with an average heterozygosity of 0.231. This value is much higher than that of Lepidopteran model insect silkworms (0.0032) and wild silkworms (0.0080) [39] (Fig 6A). Tajima’s D estimates were greater than 0 for most populations (Fig 6B), suggesting deviations from the neutral theory [40]. The north population, inhabiting predominantly flat terrains, exhibited the highest heterozygosity and the highest nucleotide diversity (π = 4.38 × 10^−3^), whereas the isolated coastal population showed the lowest π value (π = 4.08 × 10^−3^). The two southern populations, namely southwest and southeast, displayed similar θπ, which ranged between north and coastal groups (Fig 6C).

We further assessed the LD decay patterns in each geographically clustered group. LD decay varied among populations. Northern populations showed the fastest LD decay and the lowest LD levels, with r^2^ decreasing to 0.15 at only 62bp and tending to stabilize after falling to 0.08. In contrast, coastal populations had the slowest LD decay, with r^2^ decreasing to 0.15 at 441 bp. The two southern populations exhibited similar LD decay patterns, with r^2^ decreasing to 0.15 at 139 and 148bp, respectively (Fig 6D). These differences in LD decay between the north and south populations are also an indication of genetic differences between the two populations.

### Genomic signatures of positive selection

**Fig 7:**
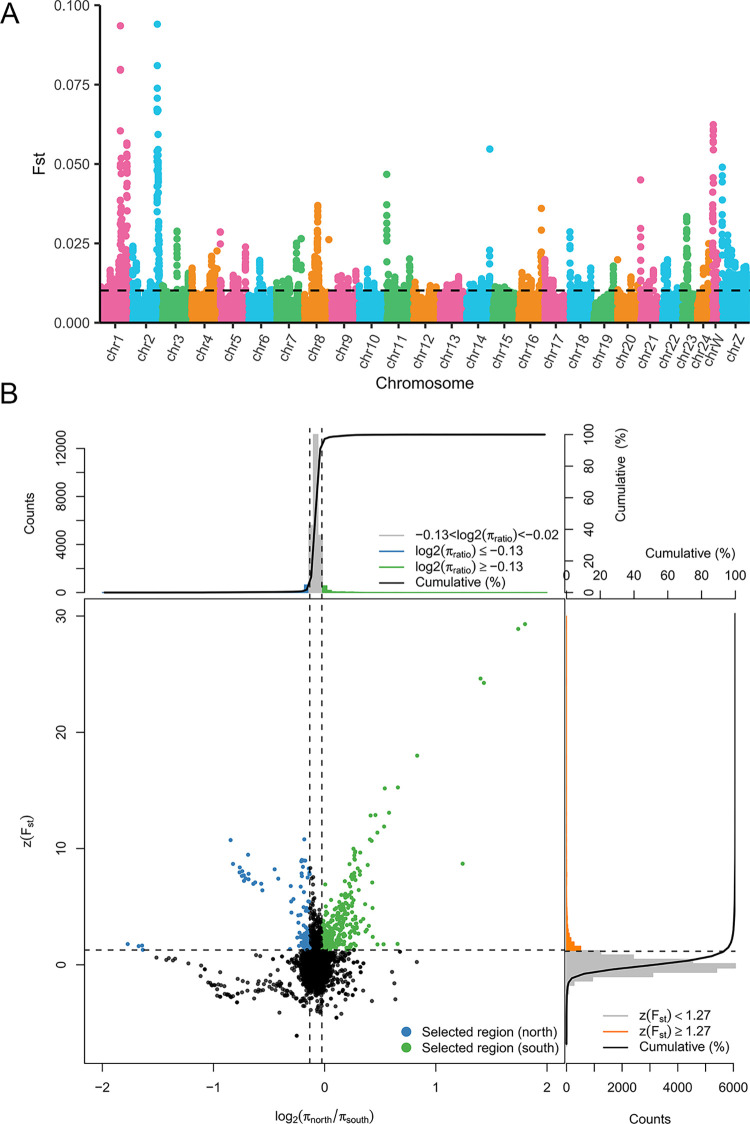
Regions of natural selection between the north and south populations. (A) Distribution of pairwise F_ST_ distances on chromosomes between north and south populations. The points above the black dotted line indicate the top 5% of F_ST_ and the region where these points are located may have been subject to natural selection (pairwise F_ST_ calculated with a window of 100 kb and a step of 10 kb). (B) The θπ ratio (north/south) and Z(F_ST_) values for north and south populations were used to identify selected regions. Selected regions were determined by the locations of the blue (north populations) and green (south populations) dots on the plot. Specifically, selected regions were those located to the left and right of the left and right vertical dashed lines, respectively (corresponding to the 5% left and right tails of the empirical θπ ratio distribution), and above the horizontal dashed line (representing the 5% right tail of the empirical Z(F_ST_) distribution).

Considering the disparate geographic ranges and climate environments between north and south populations (southwest and southeast), we mainly focused on the identification of genomic signatures of positive selection between the two populations. Using F_ST_ and θπ methods, we identified 546 selected genes in the south population and 335 selected genes in the north population (Fig 7).

**Fig 8:**
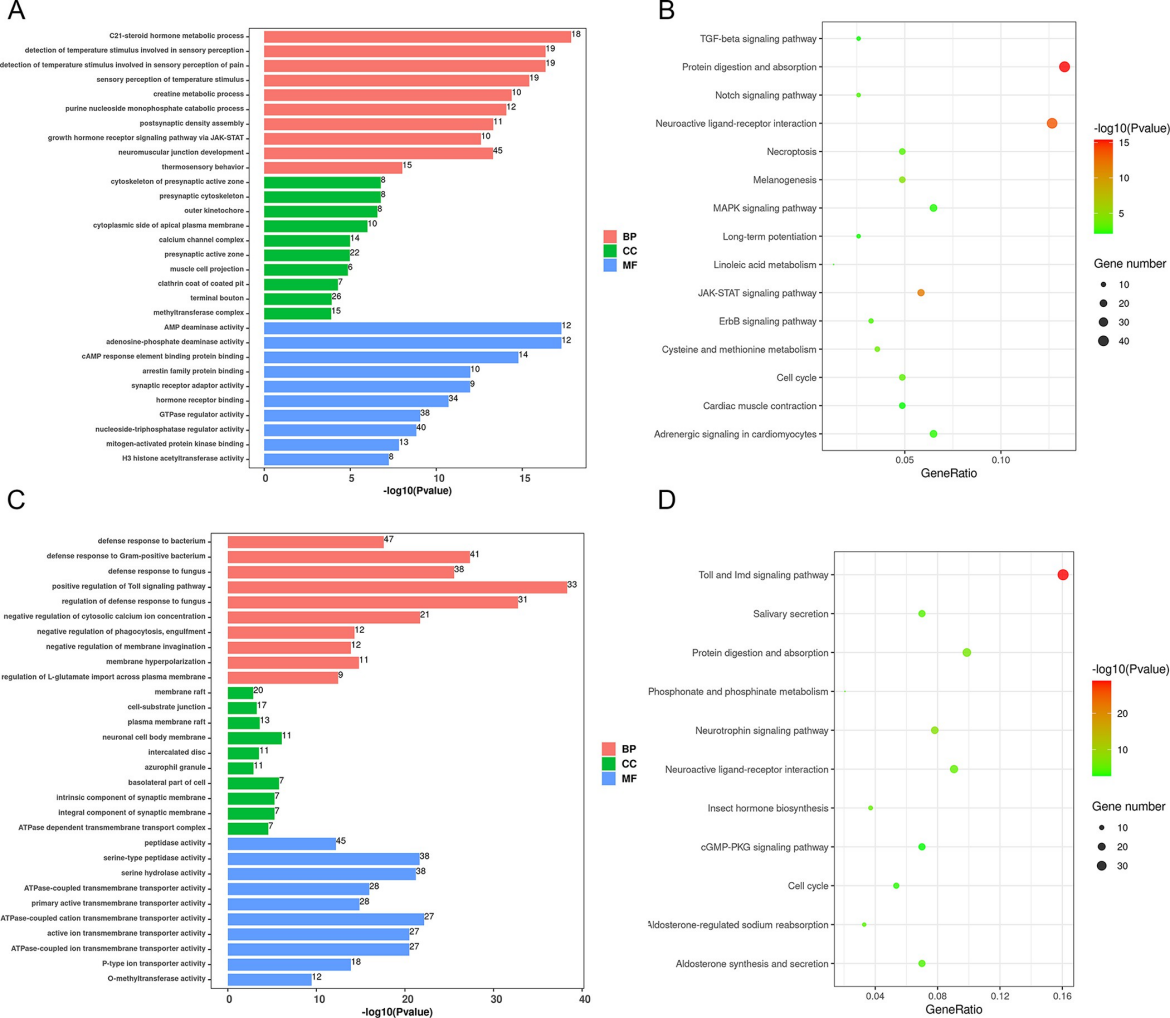
GO and KEGG enrichment analysis. (A) Enriched GO terms of selected genes in the south population. (B) Enriched KEGG pathways of selected genes in the south population. (C) Enriched GO terms of selected genes in the north population. (D) Enriched KEGG pathways of selected genes in the north population.

In the southern cabbage white butterfly, we observed many GO terms and KEGG pathways related to the growth, development, and neuromodulation of *P*. *rapae*, including the lipid and creatine metabolism (GO:0008207, P = 1.46 × 10^−18^; GO:0006600, P = 9.33 × 10^−13^), synaptic structure (GO:0099569, P = 3.07 × 10^−6^; GO:0030160, P = 9.38 × 10^−11^), the Notch signaling pathway (ko04330, P = 0.00125), the ErbB signaling pathway (ko04012, P = 0.00149), and the TGF-beta signaling pathway (ko04350, P = 0.00671) (Fig 8B and S8 Table). Furthermore, several GO terms that are associated with temperature adaptation were also significantly enriched (GO:0050961, P = 3.09 × 10^−14^; GO:0050951, P = 2.35 × 10^−13^; GO:0040040, P = 2.61 × 10^−7^) (Fig 8A and S7 Table).

In the northern cabbage white butterfly, the biological process of “defense responses” (Fig 8C and S9 Table) is highly enriched, with “defense response to bacterium” (GO:0060397, P = 3.63 × 10^−16^) and “defense response to fungus” (GO:0050832, P = 1.09 × 10^−23^) being particularly significant, suggesting the importance of resistance to pathogenic microorganisms in the adaptation of *P*. *rapae* to northern environments.

Moreover, a number of immune-related pathways were found to be significantly enriched in both the south and north populations (Fig 8D and S10 Table), including the JAK-STAT signaling pathway (ko04630, P = 9.55 × 10^−11^), MAPK signaling pathway (ko04010, P = 0.0351), Toll and Imd signaling pathway (ko04624, P = 1.21 × 10^−29^) and melanogenesis (ko04916, P = 5.41 × 10^−4^), which may indicate that the immune response may be involved in the positive selection during evolution of this butterfly.

## Discussion

Studying pests at the genomic level not only provides insights into their structure, history and adaptation, but also presents novel approaches to pest control. We have found a dense map of variation in the butterfly genomes, with large genetic admixture between different geographical groups. This is a dense variation compared to published data on model insect variation, fruit flies [41], and human variation [42]. Our F_ST_ analyses suggest that physical barriers may have contributed more to genetic differentiation than geographic distance, although we acknowledge that distance may also have played a role. Demographic history suggests that historical global temperature has profoundly affected the effective population size, with a colder climate (i.e., during glaciation) inhibiting population growth, while warmer periods (i.e., temperatures above those during glaciation but not exceeding the species’ upper thermal limit) have been associated with increased population size. Finally, we identified genes associated with positive selection.

The data from our study suggest that the genetic structure of *P*. *rapae* is not easily distinguished based on geographical location, with terrain features being more likely to influence the population’s genetic structure than geographical distance. In particular, populations inhabiting areas with closed terrain, such as Chongming (CM) with island terrain, Shandong (SD) with peninsular terrain, and southwest areas (CQ, GZ, SC, GZH) dominated by mountains and basins, show significant divergence from other populations. Like other lepidopteran pest populations [43, 44], *P*. *rapae* exhibits considerable genetic admixture among geographically connected groups, based on ADMIXTURE results and PCA analyses. This may be attributed to the rapid dispersal of *P*. *rapae*, allowing for gene flow among populations in the absence of physical barriers. The global invasion of the cabbage white butterfly is a prime example of this potential for rapid dispersal, with its geographic spread and divergence being driven by host plant diversification and human trade [9]. In China, the cabbage white butterfly is frequently transported with its host plants to different areas through national vegetable transport, resulting in frequent high gene flow between connected populations.

Our analysis also revealed that isolated populations (coastal and southwest) exhibit higher genetic differentiation and lower genetic diversity, as evidenced by higher pairwise F_ST_ and lower θπ. In contrast, populations that are less isolated (Northern China, mainly in the plains) displayed reduced differentiation, higher heterozygosity, fast LD decay, and greater genetic diversity, which is another potential evidence of high gene flow. Genetic diversity is an important factor in the survival and adaptive potential of populations and species [45]. For butterflies, higher genetic diversity has been linked to increased migratory capacity [46], which may facilitate gene flow between geographic groups. Comparatively, the degree of heterozygosity in Chinese cabbage white butterfly populations (0.231) was found to be higher than in Europe, the Americas and Australia (0.09–0.11) [9]. Heterozygosity is known to reflect the genetic diversity of a population to a certain extent [47], and a higher level of heterozygosity is associated with a stronger fitness to changing environments [48]. Moreover, gene regions with high heterozygosity have been found to be correlated with specific phenotypes [49]. Thus, we speculate that the high heterozygosity of *P*. *rapae* may be a key genetic factor for adaptation to different environments and resistance to various insecticides in China.

In exploring the historical effective population size of *P*. *rapae*, we found that it fluctuates almost in tandem with global temperature changes, with cold glacial periods leading to significant population declines and warm interglacial periods allowing recovery from population bottlenecks. This mirrored relationship between historical effective population size and temperature has been observed in many other insects [50–52]. Populations of *P*. *rapae* from northern and southeastern China are likely to exhibit a more rapid recovery from population bottlenecks and reach a higher effective population size level. Acknowledging the potential impact of population structure on our PSMC model-based inferences is crucial. Although the PSMC method operates under the assumption of panmixia, real populations frequently demonstrate structuring. Conventional population genetics methods typically overlook these structural intricacies, possibly resulting in misinterpretations or inaccuracies when inferring historical population sizes, as in these instances the inverse instantaneous coalescent rate (IICR) corresponds to the effective population size [53]. This oversight might have influenced the precision of our inferences, especially when considering regional variations.

The abundance of a species is thought to depend on its adaptation to environmental temperature and food resources [54, 55]. Studies have shown that the cabbage white butterfly has a shorter development time and higher survival rates under warm conditions [56]. Consequently, the warm subtropical climate in southeastern China and the abundant host plants in northern China provide favorable conditions for the growth and development of this butterfly. In contrast, the cabbage white butterfly in southwestern and coastal China may have suffered severe impact from the glaciations, possibly due to the closed terrain of these two regions, which could have potentially restricted the butterfly’s ability to disperse and find new habitats even during the warm interglacial period. Given the limitations associated with assuming panmixia in a structured population, further research employing models considering population structure or alternative methods could offer a more precise understanding of the historical population dynamics of *P*. *rapae*, especially concerning their responses to environmental changes.

The selected genes are identified with an outlier approach, using Fst and θπ, with the 5% right tail determining the threshold. It should be noted that if selection is rare will lead to an overestimate, and the effects of demographic history, purifying and background selection, and variation in recombination and mutation rates were not considered in this research [57, 58]. These selected genes are highly represented in development, neuromodulation and temperature sensing pathways in the south population (such as ‘C21-steroid hormone metabolism’, ‘presynaptic active zone’, ‘Notch signaling pathway’ and ‘TGF-beta signaling pathway’). Steroid hormones can influence the timing of insect molting and metamorphosis development [59]. The notch and TGF-beta signaling pathways have been found to regulate insect developmental processes, including wing vein and oogenesis differentiation in *Drosophila* [60, 61]. Moreover, the enrichment of neurosystem-related genes suggests the genetic basis of different behaviors under seasonal climate change. Neuromodulation is important for insect feeding, courtship, aggression, and postmating behaviors [62], and the number of synapses is a major determinant of behavior [63]. Evolutionary pressures induced by temperature play an important role in shaping phenotypic variation between and within species [64, 65]. Previous studies have demonstrated that the abundance of many metabolites in *P*. *rapae* and warming-induced key biochemical pathways have changed significantly in warming environments [66]. In a study of the model insect, *Drosophila*, those living in the tropics were found to lack genetic variation in tolerance to extreme environments such as dry and cold [67]. Compared to north population, these selected genes may be related to the greater plasticity in terms of temperature, as well as the shorter development time, more annual generations, and strong reproductive capabilities of *P*. *rapae* in south population.

Another important characteristic of positive selection during evolution in *P*. *rapae* is a variety of selected genes involved in defense responses in northern China. The climate disparity between North and South China results in differing pathogenic microorganisms. The enrichment of genes involved in defense responses is a possible indication of adaptation to the local environment. Delving deeper into these genes may reveal the underlying mechanisms of the cabbage white butterfly’s response to external stimuli. Furthermore, we identified several pathways related to immune response in both the south and north population, including the JAK-STAT signaling pathway, MAPK signaling pathway, and melanogenesis. Unlike mammals, insects rely on their innate immune system to protect themselves from foreign substances and microorganisms [68]. The JAK-STAT signaling pathway is mediated by cytokines to induce the expression of multiple immune genes [69], while the MAPK signaling pathway regulates the expression of antimicrobial peptides and the intensity of immune response [70]. Melanin is an important immune effector, capable of killing and eliminating pathogens [71, 72]. These enriched pathways that are essential for the immune response of the insect display a strong geographical dependence, which could potentially be attributed to selection pressure from insecticides and parasites. As a result, the cabbage white butterfly possesses rapidly evolving genes related to its immune system which may facilitate its rapid adaptation to the local environment.

## Conclusion

Our research has focused on the widespread populations of *P*. *rapae* in China, utilizing population genomics approaches to provide insights into the population differentiation, structure, demographic history and adaptive evolution of this species. We reveal significant genomic variation and heterozygosity across the entire genome of *P*. *rapae*. In addition, we characterized the population genetic structure of *P*. *rapae* and uncovered the intrinsic relationships among different geographical groups in China. Geographical distance did not prevent genetic communication between *P*. *rapae* populations, while terrain barriers may have a more profound effect on the population divergence of this butterfly. The effective size of the population is strongly influenced by global temperatures. Additionally, we identified the selected genes under different climates to address the potential mechanisms of local adaptation, which will promote a better understanding of how the environment has shaped patterns of genetic variation and rapid evolution that are triggered by selective pressures.

## Supporting information

S1 Fig Cross-validation errors for different K values. (TIF)

S2 Fig Scatter plot principal components 1 versus 3 (PC1 versus PC2). (TIF)

S3 Fig Scatter plot principal components 3 versus 4 (PC3 versus PC4). (TIF)

S4 Fig Demographic history of *P*. *rapae* in four populations. The clear red line indicates the effective population size measured by the scaled mutation rate per generation (0.2×10^−8^), and the thin red line illustrates the PSMC estimates for 20 rounds of bootstrap.
(TIF)

S1 Table Population geographic information and strain classification of *P*. *rapae*. (PDF)

S2 Table The concentration of DNA sample of *P*. *rapae*. (PDF)

S3 Table Statistics of genomic sequencing data of *P*. *rapae*. (PDF)

S4 Table Summary of mapping and coverage rate of *P*. *rapae*. (PDF)

S5 Table SNP annotation. (PDF)

S6 Table Pairwise F_ST_ distances between *P*. *rapae* populations. (PDF)

S7 Table Enriched GO terms in south population. (PDF)

S8 Table Enriched KEGG pathway of selected genes in south population. (PDF)

S9 Table Enriched GO terms in north population. (PDF)

S10 Table Enriched KEGG pathway of selected genes in north population. (PDF)
