# Supplementary figures and images for: Population genomics provides insights into the genetic diversity and adaptation of the Pieris rapae in China
Source: PLoS One. 2023 Nov 16;18(11):e0294521. doi: 10.1371/journal.pone.0294521 (PMC10653512; doi:10.1371/journal.pone.0294521)

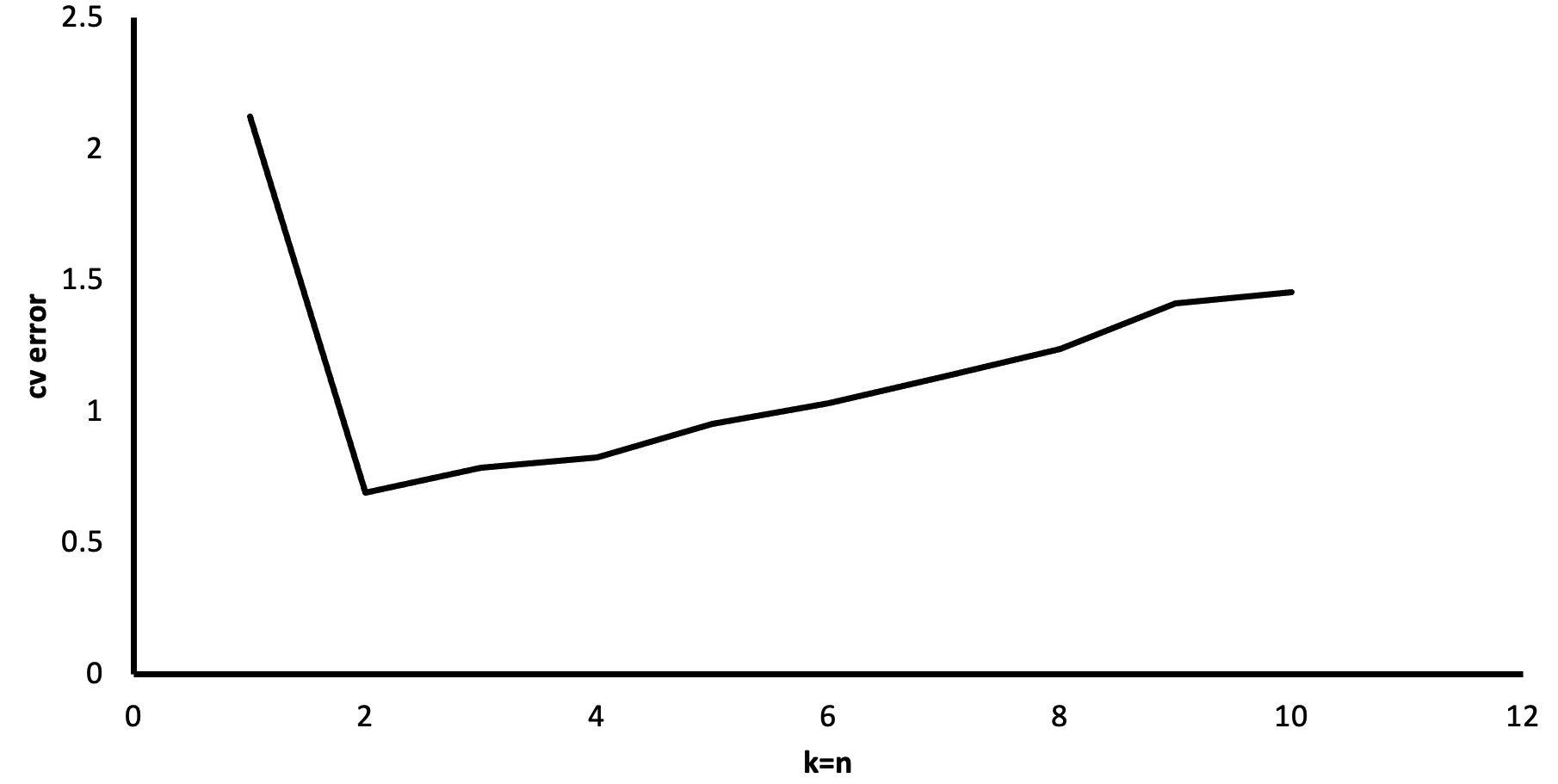

Supplement: S1 Fig — (TIF) [file pone.0294521.s001.tif]

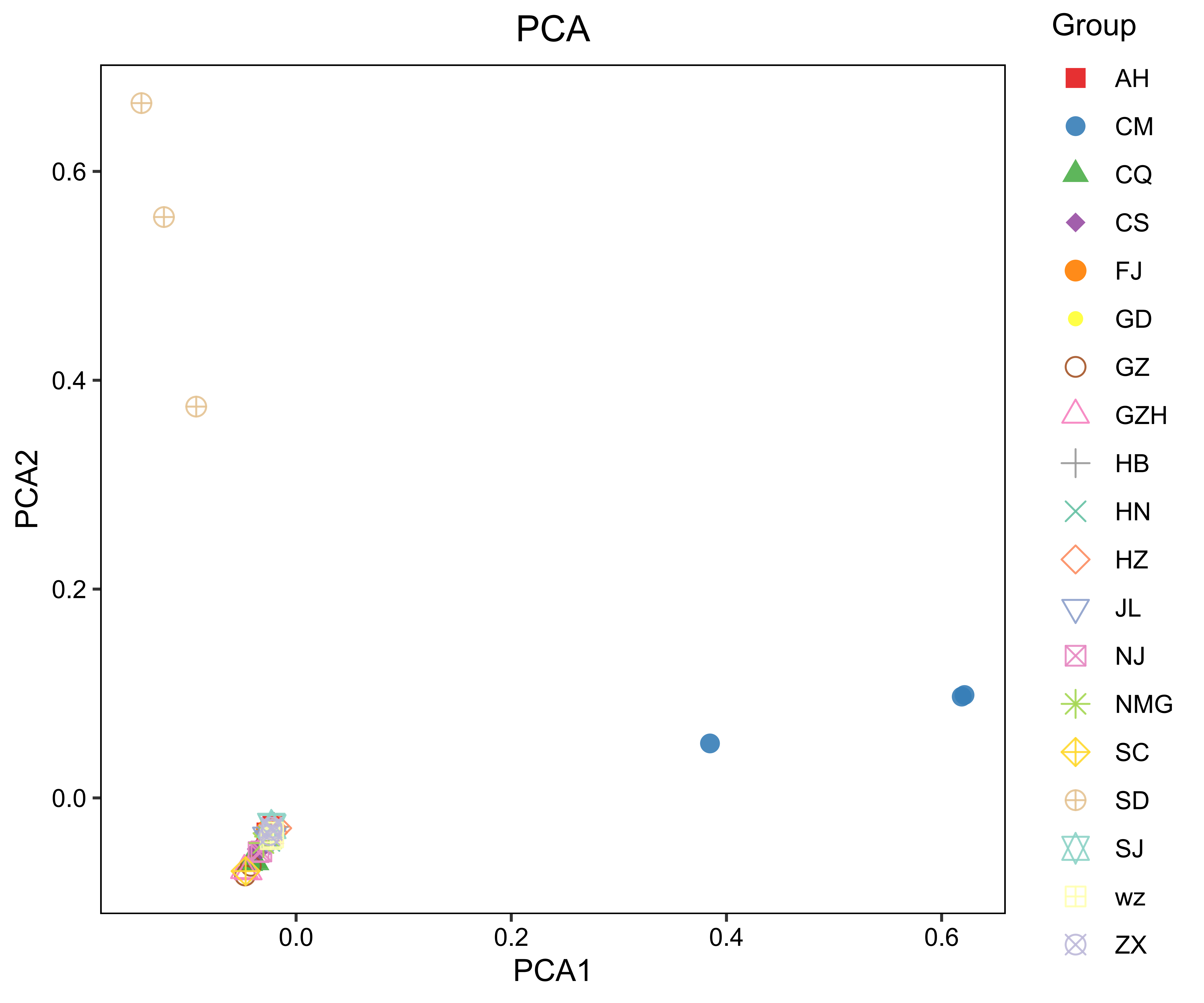

Supplement: S2 Fig — (TIF) [file pone.0294521.s002.tif]

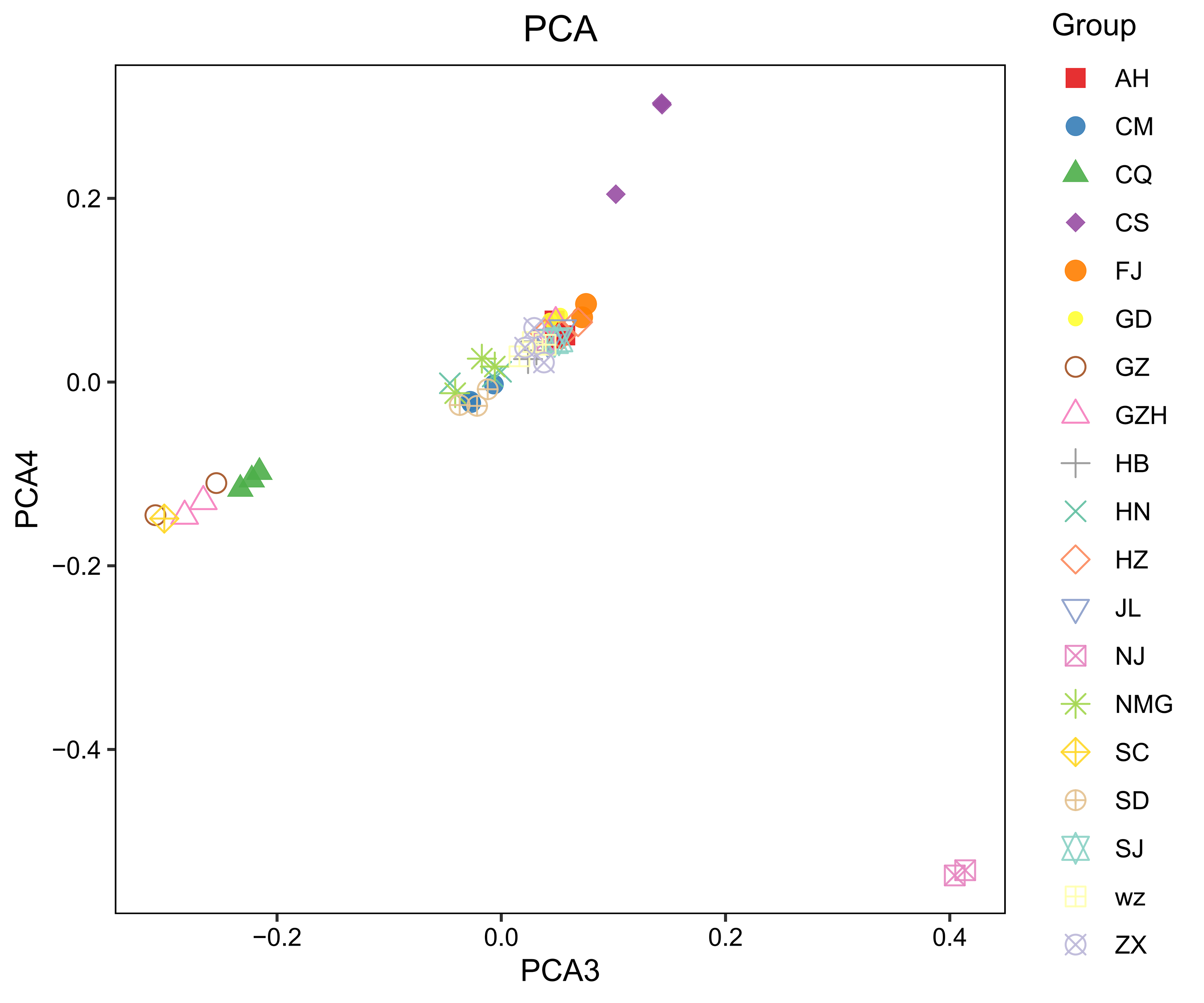

Supplement: S3 Fig — (TIF) [file pone.0294521.s003.tif]
