# Supplementary material for: Population genomics provides insights into the genetic diversity and adaptation of the Pieris rapae in China
Source: PLoS One. 2023 Nov 16;18(11):e0294521. doi: 10.1371/journal.pone.0294521 (PMC10653512; doi:10.1371/journal.pone.0294521)
Supplement: S1 Table — (PDF) [file pone.0294521.s005.pdf]

Table S1 Population geographic information and strain classification of *P. rapae*

Sample	Location	Longitude	Latitude	Population	Colonies
NMG1	Neimenggu	111.765617	40.817498	north	
NMG2	Neimenggu	111.765617	40.817498	north	
NMG3	Neimenggu	111.765617	40.817498	north	
HB1	Hebei	114.468664	38.037057	north	
HB2	Hebei	114.468664	38.037057	north	
HB3	Hebei	114.468664	38.037057	north	
HN1	Henan	113.753602	34.765515	north	
HN2	Henan	113.753602	34.765515	north	
HN3	Henan	113.753602	34.765515	north	18
JL1	Siping,Jilin	124.350398	43.166419	north	
JL2	Siping,Jilin	124.350398	43.166419	north	
JL3	Siping,Jilin	124.350398	43.166419	north	
wz1	Shanxi	112.899137	35.617221	north	
wz2	Shanxi	112.899137	35.617221	north	
wz3	Shanxi	112.899137	35.617221	north	
CS1	Beijing	116.29905	40.1541	north	
CS2	Beijing	116.29905	40.1541	north	
CS3	Beijing	116.29905	40.1541	north	
CM1	Chongming,Shanghai	121.397417	31.623587	coastal	
CM2	Chongming,Shanghai	121.397417	31.623587	coastal	
CM3	Chongming,Shanghai	121.397417	31.623587	coastal	
SD1	Shandong	117.020359	36.66853	coastal	
SD2	Shandong	117.020359	36.66853	coastal	10
SD3	Shandong	117.020359	36.66853	coastal	
FJ1	Fujian	119.295144	26.100779	coastal	
FJ2	Fujian	119.295144	26.100779	coastal	
GD1	Guangdong	113.26653	23.132191	coastal	
GD3	Guangdong	113.26653	23.132191	coastal	
HZ1	Hagnzhou,Zhejiang	120.15507	30.274084	southeast	
HZ2	Hagnzhou,Zhejiang	120.15507	30.274084	southeast	
HZ3	Hagnzhou,Zhejiang	120.15507	30.274084	southeast	
NJ1	Nanjing,Jiangsu	118.796877	32.060255	southeast	
NJ2	Nanjing,Jiangsu	118.796877	32.060255	southeast	
NJ3	Nanjing,Jiangsu	118.796877	32.060255	southeast	11
AH1	Anhui	117.284922	31.861184	southeast	
AH2	Anhui	117.284922	31.861184	southeast	
AH3	Anhui	117.284922	31.861184	southeast	
SJ4	Songjiang,Shanghai	121.227747	31.032243	southeast	
SJ5	Songjiang,Shanghai	121.227747	31.032243	southeast	

ZX1	Zixi,Jiangxi	117.060264	27.706102	southwest	
ZX2	Zixi,Jiangxi	117.060264	27.706102	southwest	
ZX3	Zixi,Jiangxi	117.060264	27.706102	southwest	
GZH1	Ganzhou,Jiangxi	114.935029	25.831829	southwest	
GZH2	Ganzhou,Jiangxi	114.935029	25.831829	southwest	
GZH3	Ganzhou,Jiangxi	114.935029	25.831829	southwest	12
GZ1	Gunzhou	106.70741	26.598194	southwest	
GZ3	Gunzhou	106.70741	26.598194	southwest	
CQ1	Chongqing	106.551556	29.563009	southwest	
CQ2	Chongqing	106.551556	29.563009	southwest	
CQ3	Chongqing	106.551556	29.563009	southwest	
SC1	Sichuan	104.075931	30.651651	southwest	
