# Supplementary material for: Population genomics provides insights into the genetic diversity and adaptation of the Pieris rapae in China
Source: PLoS One. 2023 Nov 16;18(11):e0294521. doi: 10.1371/journal.pone.0294521 (PMC10653512; doi:10.1371/journal.pone.0294521)
Supplement: S2 Table — (PDF) [file pone.0294521.s006.pdf]

Table S2 The concentration of DNA sample of *P. rapae*

Sample	concentration(ng/ul)	volume(ul)	total(ug)	conclusion
HZ1	21.351	60	1.28106	A
HZ2	11.897	60	0.71382	A
HZ3	14.389	60	0.86334	A
CM1	66.71	60	4.0026	A
CM2	36.747	60	2.20482	A
CM3	21.055	60	1.2633	A
SD1	37.244	60	2.23464	A
SD2	17.325	60	1.0395	A
SD3	7.344	60	0.44064	A
GZ1	66.18	60	3.9708	A
GZ3	23.19	60	1.3914	A
NJ1	22.262	60	1.33572	A
NJ2	6.742	60	0.40452	A
NJ3	9.811	60	0.58866	A
AH1	18.711	60	1.12266	A
AH2	19.23	60	1.1538	A
AH3	25.111	60	1.50666	A
ZX1	33.307	60	1.99842	A
ZX2	20.619	60	1.23714	A
ZX3	15.469	60	0.92814	A
GZH1	58.154	60	3.48924	A
GZH2	12.063	60	0.72378	A
GZH3	25.764	60	1.54584	A
NMG1	13.086	60	0.78516	A
NMG2	19.768	60	1.18608	A
NMG3	8.834	60	0.53004	A
HB1	96.585	60	5.7951	A
HB2	24.942	60	1.49652	A
HB3	22.735	60	1.3641	A
FJ1	10.281	60	0.61686	A
FJ2	29.987	60	1.79922	A

CQ1	13.518	60	0.81108	A
CQ2	17.375	60	1.0425	A
CQ3	20.895	60	1.2537	A
GD1	33.616	79	2.65566	A
GD3	20.088	60	1.20528	A
HN1	31.964	79	2.52516	A
HN2	21.07	60	1.2642	A
HN3	12.278	60	0.73668	A
SC1	27.411	60	1.64466	A
JL1	23.302	60	1.39812	A
JL2	72.514	60	4.35084	A
JL3	44.206	60	2.65236	A
wz1	25.427	60	1.52562	A
wz2	20.001	60	1.20006	A
wz3	34.587	60	2.07522	A
CS1	8.799	60	0.52794	A
CS2	17.725	60	1.0635	A
CS3	29.559	60	1.77354	A
SJ4	14.558	60	0.87348	A
SJ5	36.099	60	2.16594	A

Note: The test conclusion A indicates that the sample quality meets the quality requirements of library construction sequencing, and the total amount meets the needs of 1 or more database construction.
