# Supplementary material for: Population genomics provides insights into the genetic diversity and adaptation of the Pieris rapae in China
Source: PLoS One. 2023 Nov 16;18(11):e0294521. doi: 10.1371/journal.pone.0294521 (PMC10653512; doi:10.1371/journal.pone.0294521)
Supplement: S3 Table — (PDF) [file pone.0294521.s007.pdf]

Table S3 Statistics of genomic sequencing data of *P. rapae*

SampleID	Raw_Reads	Clean_Reads	Raw_Base	Clean_Base	Effective rate(%)	Q20(%)	Q30(%)	GC(%)
AH1	21,281,440	21,274,744	3,192,216,000	3,186,637,302	99.83%	98.53	95.1	33.79
AH2	21,512,896	21,505,122	3,226,934,400	3,219,762,061	99.78%	98.49	95.03	33.18
AH3	23,702,038	23,695,124	3,555,305,700	3,548,820,490	99.82%	98.37	94.67	33.04
CM1	49,166,652	49,157,636	7,374,997,800	7,368,594,358	99.91%	97.84	93.39	37.46
CM2	51,028,212	51,018,804	7,654,231,800	7,646,028,318	99.89%	97.84	93.37	37.73
CM3	51,867,828	51,857,488	7,780,174,200	7,771,844,845	99.89%	97.97	93.63	38.07
CQ1	22,415,180	22,408,456	3,362,277,000	3,357,518,041	99.86%	98.53	95.17	33.38
CQ2	27,643,900	27,635,054	4,146,585,000	4,139,935,917	99.84%	98.47	95.01	33.11
CQ3	53,601,298	53,584,384	8,040,194,700	8,030,166,128	99.88%	97.83	93.73	47.92
CS1	22,475,566	22,467,978	3,371,334,900	3,367,072,057	99.87%	98.5	95.04	33.19
CS2	22,088,130	22,076,530	3,313,219,500	3,307,402,378	99.82%	98.5	95.02	33.2
CS3	20,293,026	20,288,226	3,043,953,900	3,039,797,079	99.86%	97.44	92.32	34.2
FJ1	20,855,662	20,839,312	3,128,349,300	3,121,427,566	99.78%	98.52	95.13	33.02
FJ2	21,298,860	21,288,078	3,194,829,000	3,189,646,363	99.84%	98.45	94.95	32.76
GD1	26,825,298	26,808,594	4,023,794,700	4,015,461,736	99.79%	98.4	94.88	32.91
GD3	23,256,810	23,250,092	3,488,521,500	3,482,990,443	99.84%	98.5	95.04	33.54
GZ1	61,138,560	61,128,436	9,170,784,000	9,160,142,985	99.88%	97.96	93.91	51.01
GZ3	50,457,646	50,451,246	7,568,646,900	7,558,733,609	99.87%	98.25	94.69	49.99
GZH1	78,095,504	78,081,832	11,714,325,600	11,698,285,493	99.86%	97.97	93.83	47.26
GZH2	23,096,242	23,089,008	3,464,436,300	3,459,615,358	99.86%	98.55	95.16	33.57
GZH3	24,119,564	24,109,218	3,617,934,600	3,611,772,112	99.83%	98.34	94.72	34.24
HB1	23,388,408	23,385,378	3,508,261,200	3,504,440,842	99.89%	98.39	94.89	41.44
HB2	43,069,400	43,057,260	6,460,410,000	6,451,527,060	99.86%	98.04	93.86	34.61
HB3	22,150,300	22,138,268	3,322,545,000	3,316,865,026	99.83%	98.31	94.59	33.81
HN1	40,042,518	40,029,180	6,006,377,700	5,996,299,726	99.83%	97.91	93.57	37.65
HN2	20,037,778	20,030,504	3,005,666,700	3,000,936,156	99.84%	98.38	94.69	33.56
HN3	20,245,348	20,234,710	3,036,802,200	3,031,671,040	99.83%	98.43	94.89	33.69
HZ1	27,120,106	27,105,214	4,068,015,900	4,060,287,423	99.81%	98.5	95.06	33.6
HZ2	23,209,782	23,201,828	3,481,467,300	3,475,496,695	99.83%	98.37	94.77	33.59
HZ3	27,698,214	27,671,768	4,154,732,100	4,145,628,879	99.78%	98.65	95.41	33.81

JL1	22,269,332	22,261,564	3,340,399,800	3,335,039,845	99.84%	98.46	94.94	33.26
JL2	50,824,344	50,812,306	7,623,651,600	7,616,636,120	99.91%	98.06	93.83	37.59
JL3	56,967,862	56,958,516	8,545,179,300	8,537,766,264	99.91%	97.69	93.05	38.18
NJ1	26,751,478	26,743,284	4,012,721,700	4,006,501,634	99.84%	98.56	95.19	34.01
NJ2	21,930,294	21,924,210	3,289,544,100	3,283,178,974	99.81%	98.22	94.25	34.21
NJ3	22,855,722	22,837,360	3,428,358,300	3,420,242,579	99.76%	98.39	94.78	34.01
NMG1	21,219,854	21,212,702	3,182,978,100	3,178,372,073	99.86%	98.46	94.96	33.37
NMG2	21,159,436	21,148,342	3,173,915,400	3,168,194,820	99.82%	98.5	95.12	34.13
NMG3	26,977,686	26,967,484	4,046,652,900	4,040,627,436	99.85%	98.34	94.65	33.38
SC1	21,653,064	21,644,974	3,247,959,600	3,242,838,400	99.84%	98.43	94.88	33.6
SD1	22,392,618	22,384,852	3,358,892,700	3,353,598,865	99.84%	98.46	94.96	33.72
SD2	22,604,756	22,594,924	3,390,713,400	3,384,798,600	99.83%	98.24	94.32	33.41
SD3	24,563,518	24,540,868	3,684,527,700	3,675,902,374	99.77%	98.44	94.95	33.97
SJ4	27,156,092	27,134,716	4,073,413,800	4,064,848,614	99.79%	97.56	92.68	34.12
SJ5	40,898,450	40,883,900	6,134,767,500	6,124,176,095	99.83%	97.55	92.61	35.31
ZX1	22,971,434	22,963,326	3,445,715,100	3,440,122,400	99.84%	98.46	94.97	34.47
ZX2	21,636,754	21,621,524	3,245,513,100	3,238,932,715	99.80%	98.23	94.36	33.38
ZX3	22,584,740	22,575,848	3,387,711,000	3,381,293,824	99.81%	98.38	94.8	33.7
wz1	25,945,580	25,933,966	3,891,837,000	3,883,827,110	99.79%	98.23	94.4	33.9
wz2	20,783,534	20,777,444	3,117,530,100	3,112,994,010	99.85%	98.48	95.04	33.32
wz3	21,218,920	21,208,250	3,182,838,000	3,177,766,916	99.84%	98.49	95.05	33.07
