# Supplementary material for: Population genomics provides insights into the genetic diversity and adaptation of the Pieris rapae in China
Source: PLoS One. 2023 Nov 16;18(11):e0294521. doi: 10.1371/journal.pone.0294521 (PMC10653512; doi:10.1371/journal.pone.0294521)
Supplement: S4 Table — (PDF) [file pone.0294521.s008.pdf]

Table S4 Summary of mapping and coverage rate of *P. rapae*

Sample	Clean_reads	mapped_reads	mapping_rate	Average_depth	Coverage_1X	Coverage_4X
AH1	17132181	16631881	97.08%	8.72	93.19%	84.90%
AH2	17,291,336	15,772,155	91.21%	8.19	92.78%	83.79%
AH3	19,901,298	16,959,795	85.22%	8.69	93.10%	84.78%
CM1	45,479,484	19,338,575	42.52%	9.78	92.66%	86.38%
CM2	47,311,687	17,900,542	37.84%	8.94	93.10%	85.12%
CM3	48,568,715	16,878,771	34.75%	8.25	92.35%	81.45%
CQ1	17,666,111	17,404,825	98.52%	9.10	92.84%	85.70%
CQ2	21,637,771	21,307,103	98.47%	11.16	93.61%	88.04%
CQ3	49,306,650	20,321,138	41.21%	10.63	93.46%	87.22%
CS1	17,640,551	17,360,179	98.41%	9.09	93.03%	85.78%
CS2	17,287,261	17,013,487	98.42%	8.90	93.23%	85.42%
CS3	16,894,844	15,896,155	94.09%	8.31	93.04%	83.74%
FJ1	16,931,567	14,962,729	88.37%	7.73	92.79%	81.87%
FJ2	17,167,343	15,534,935	90.49%	8.05	92.49%	83.15%
GD1	22,340,080	19,017,192	85.13%	9.64	93.22%	86.35%
GD3	18,050,948	17,657,912	97.82%	9.25	93.34%	85.05%
GZ1	58,183,244	14,820,583	25.47%	7.62	92.29%	80.99%
GZ3	47,191,996	15,626,827	33.11%	8.10	92.47%	83.00%
GZH1	74,544,025	13,639,109	18.30%	6.65	92.05%	77.21%
GZH2	18,432,857	18,155,839	98.50%	9.53	93.38%	85.64%
GZH3	19,464,427	19,069,325	97.97%	10.01	93.29%	86.05%
HB1	21,600,904	8,521,103	39.45%	4.19	89.09%	53.03%
HB2	37,901,276	26,570,708	70.11%	13.77	94.21%	90.42%
HB3	18,169,250	17,892,556	98.48%	9.38	93.20%	85.23%
HN1	34,573,201	25,379,289	73.41%	13.27	94.12%	90.17%
HN2	15,962,709	14,995,551	93.94%	7.85	92.94%	80.90%
HN3	16,064,910	15,566,295	96.90%	8.16	92.93%	82.23%
HZ1	21,728,818	21,395,423	98.47%	11.22	93.24%	88.20%
HZ2	18,342,159	18,062,143	98.47%	9.48	93.49%	86.36%
HZ3	21,014,102	20,244,528	96.34%	10.58	93.05%	87.19%
JL1	17,635,959	17,348,481	98.37%	9.09	93.44%	84.97%

JL2	47,450,208	15,966,227	33.65%	8.33	93.13%	83.23%
JL3	53,332,232	17,838,711	33.45%	8.58	92.99%	84.29%
NJ1	21,964,657	18,971,589	86.37%	9.88	93.10%	86.86%
NJ2	18,213,832	14,969,459	82.19%	7.73	92.95%	80.90%
NJ3	18,496,465	16,786,583	90.76%	8.73	93.32%	83.66%
NMG1	17,147,604	16,884,979	98.47%	8.86	92.84%	85.12%
NMG2	17,232,656	16,087,314	93.35%	8.40	92.92%	83.81%
NMG3	21,947,535	21,603,870	98.43%	11.33	93.61%	88.66%
SC1	17,137,056	16,373,027	95.54%	8.55	93.06%	83.36%
SD1	17,623,728	17,350,373	98.45%	9.08	92.90%	84.50%
SD2	17,660,144	17,400,361	98.53%	9.11	92.68%	85.36%
SD3	19,141,723	18,230,089	95.24%	9.54	92.25%	84.94%
SJ4	22,827,194	20,667,551	90.54%	10.81	93.15%	88.02%
SJ5	36,212,325	27,525,964	76.01%	14.30	93.63%	90.01%
wz1	21,164,650	19,920,434	94.12%	10.41	93.41%	87.74%
wz2	16,725,499	16,195,364	96.83%	8.47	93.03%	83.26%
wz3	17,487,725	14,821,484	84.75%	7.54	92.79%	80.11%
ZX1	18,422,693	17,273,247	93.76%	9.05	93.42%	84.78%
ZX2	17,158,022	16,870,736	98.33%	8.84	93.35%	84.26%
ZX3	17,994,586	17,698,432	98.35%	9.29	93.38%	85.07%
