# Supplementary material for: Population genomics provides insights into the genetic diversity and adaptation of the Pieris rapae in China
Source: PLoS One. 2023 Nov 16;18(11):e0294521. doi: 10.1371/journal.pone.0294521 (PMC10653512; doi:10.1371/journal.pone.0294521)
Supplement: S5 Table — (PDF) [file pone.0294521.s009.pdf]

Table S5 SNP annotation

	category	Number of SNPs
	intronic	2500447
	intergenic	1255191
exonic	synonymous	365873
exonic	nonsynonymous	92680
exonic	stopgain	400
exonic	stoploss	57
	3'UTR	134188
	upstream	129732
	downstream	114545
	upstream/downstream	21439
	5'UTR	38846
	ncRNA_intronic	33161
	ncRNA_exonic	9135
	splicing	198
	total	4696801
