# Supplementary material for: Population genomics provides insights into the genetic diversity and adaptation of the Pieris rapae in China
Source: PLoS One. 2023 Nov 16;18(11):e0294521. doi: 10.1371/journal.pone.0294521 (PMC10653512; doi:10.1371/journal.pone.0294521)
Supplement: S6 Table — (PDF) [file pone.0294521.s010.pdf]

Table S6 Pairwise F_{ST} distances between *P. rapae* populations

	north	southeast	southwest	coastal
north	-			
southeast	0.073	-		
southwest	0.069	0.059	-	
coastal	0.076	0.066	0.098	-
