# Supplementary material for: Population genomics provides insights into the genetic diversity and adaptation of the Pieris rapae in China
Source: PLoS One. 2023 Nov 16;18(11):e0294521. doi: 10.1371/journal.pone.0294521 (PMC10653512; doi:10.1371/journal.pone.0294521)
Supplement: S7 Table — (PDF) [file pone.0294521.s011.pdf]

Table S7 Enriched GO terms in south population

GO_accession	class	Description	count	Pvalue	Corrected_Pvalue
GO:0008207	BP	C21-steroid hormone metabolic process	18	1.46E-18	3.49E-15
GO:0050961	BP	detection of temperature stimulus involved in sensory perception	19	4.52E-17	3.09E-14
GO:0050965	BP	detection of temperature stimulus involved in sensory perception of pain	19	4.52E-17	3.09E-14
GO:0050951	BP	sensory perception of temperature stimulus	19	3.93E-16	2.35E-13
GO:0006600	BP	creatine metabolic process	10	4.10E-15	9.33E-13
GO:0009128	BP	purine nucleoside monophosphate catabolic process	12	8.45E-15	1.68E-12
GO:0097107	BP	postsynaptic density assembly	11	4.86E-14	6.62E-12
GO:0060397	BP	growth hormone receptor signaling pathway via JAK-STAT	10	2.53E-13	2.81E-11
GO:0007528	BP	neuromuscular junction development	45	4.98E-14	6.62E-12
GO:0040040	BP	thermosensory behavior	15	9.27E-09	2.61E-07
GO:0003876	MF	AMP deaminase activity	12	5.31E-18	5.08E-15
GO:0047623	MF	adenosine-phosphate deaminase activity	12	5.31E-18	5.08E-15
GO:0008140	MF	cAMP response element binding protein binding	14	1.67E-15	7.24E-13
GO:1990763	MF	arrestin family protein binding	10	1.06E-12	9.38E-11
GO:0030160	MF	synaptic receptor adaptor activity	9	1.10E-12	9.38E-11
GO:0051427	MF	hormone receptor binding	34	1.93E-11	1.15E-09
GO:0030695	MF	GTPase regulator activity	38	8.49E-10	3.30E-08
GO:0060589	MF	nucleoside-triphosphatase regulator activity	40	1.42E-09	5.24E-08
GO:0051019	MF	mitogen-activated protein kinase binding	13	1.48E-08	3.95E-07
GO:0010484	MF	H3 histone acetyltransferase activity	8	5.69E-08	1.20E-06
GO:0048788	CC	cytoskeleton of presynaptic active zone	8	1.66E-07	3.07E-06
GO:0099569	CC	presynaptic cytoskeleton	8	1.66E-07	3.07E-06
GO:0000940	CC	outer kinetochore	8	2.67E-07	4.65E-06
GO:0098592	CC	cytoplasmic side of apical plasma membrane	10	9.95E-07	1.44E-05
GO:0034704	CC	calcium channel complex	14	1.02E-05	0.00011962
GO:0048786	CC	presynaptic active zone	22	1.05E-05	0.00012231
GO:0036194	CC	muscle cell projection	6	1.39E-05	0.00015566
GO:0030132	CC	clathrin coat of coated pit	7	5.23E-05	0.00049021
GO:0043195	CC	terminal bouton	26	0.00012	0.00098749
GO:0034708	CC	methyltransferase complex	15	0.000129	0.00106395
