# Supplementary material for: Population genomics provides insights into the genetic diversity and adaptation of the Pieris rapae in China
Source: PLoS One. 2023 Nov 16;18(11):e0294521. doi: 10.1371/journal.pone.0294521 (PMC10653512; doi:10.1371/journal.pone.0294521)
Supplement: S8 Table — (PDF) [file pone.0294521.s012.pdf]

Table S8 Enriched KEGG pathway of selected genes in south population

ID	Kegg_pathway	GeneRatio	Pvalue	Corrected_P-value	Gene_number
ko04080	Neuroactive ligand-receptor interaction	0.126623	2.56E-14	1.95E-12	39
ko04010	MAPK signaling pathway	0.064935	0.005366	0.03511	20
ko04330	Notch signaling pathway	0.025974	0.001246	0.010567	8
ko04012	ErbB signaling pathway	0.032468	0.001489	0.01218	10
ko04630	JAK-STAT signaling pathway	0.058442	1.67E-12	9.55E-11	18
ko04350	TGF-beta signaling pathway	0.025974	0.006711	0.040442	8
ko04110	Cell cycle	0.048701	0.000589	0.006428	15
ko04217	Necroptosis	0.048701	0.001017	0.009398	15
ko04916	Melanogenesis	0.048701	2.83E-05	0.000541	15
ko04261	Adrenergic signaling in cardiomyocytes	0.064935	0.004573	0.031734	20
ko04720	Long-term potentiation	0.025974	0.006711	0.040442	8
ko04974	Protein digestion and absorption	0.133117	4.09E-16	4.68E-14	41
ko04260	Cardiac muscle contraction	0.048701	0.007418	0.043554	15
ko00591	Linoleic acid metabolism	0.012987	0.005273	0.03511	4
ko00270	Cysteine and methionine metabolism	0.035714	0.000178	0.002266	11
