# Supplementary material for: Population genomics provides insights into the genetic diversity and adaptation of the Pieris rapae in China
Source: PLoS One. 2023 Nov 16;18(11):e0294521. doi: 10.1371/journal.pone.0294521 (PMC10653512; doi:10.1371/journal.pone.0294521)
Supplement: S9 Table — (PDF) [file pone.0294521.s013.pdf]

Table S9 Enriched GO terms in north population

GO_accession	class	Description	count	Pvalue	Corrected_Pvalue
GO:0042742	BP	defense response to bacterium	47	2.53E-18	3.63E-16
GO:0050830	BP	defense response to Gram-positive bacterium	41	4.51E-28	2.91E-25
GO:0050832	BP	defense response to fungus	38	2.81E-26	1.09E-23
GO:0045752	BP	positive regulation of Toll signaling pathway	33	4.80E-39	1.86E-35
GO:1900150	BP	regulation of defense response to fungus	31	1.76E-33	1.36E-30
GO:0051481	BP	negative regulation of cytosolic calcium ion concentration	21	1.99E-22	4.95E-20
GO:0060101	BP	negative regulation of phagocytosis, engulfment	12	5.18E-15	5.58E-13
GO:1905154	BP	negative regulation of membrane invagination	12	1.26E-14	1.26E-12
GO:0060081	BP	membrane hyperpolarization	11	1.58E-15	1.75E-13
GO:0002036	BP	regulation of L-glutamate import across plasma membrane	9	3.55E-13	3.12E-11
GO:0045121	CC	membrane raft	20	0.0013541	0.01254673
GO:0030055	CC	cell-substrate junction	17	0.0005322	0.00604499
GO:0044853	CC	plasma membrane raft	13	0.0002532	0.00333507
GO:0032809	CC	neuronal cell body membrane	11	8.16E-07	2.09E-05
GO:0014704	CC	intercalated disc	11	0.0002918	0.00377936
GO:0042582	CC	azurophil granule	11	0.0012634	0.01208179
GO:1990794	CC	basolateral part of cell	7	1.64E-06	3.82E-05
GO:0099240	CC	intrinsic component of synaptic membrane	7	5.40E-06	0.00011313
GO:0099699	CC	integral component of synaptic membrane	7	5.40E-06	0.00011313
GO:0098533	CC	ATPase dependent transmembrane transport complex	7	2.78E-05	0.00045279
GO:0008233	MF	peptidase activity	45	6.18E-13	5.30E-11
GO:0008236	MF	serine-type peptidase activity	38	2.23E-22	5.09E-20
GO:0017171	MF	serine hydrolase activity	38	5.82E-22	1.13E-19
GO:0042626	MF	ATPase-coupled transmembrane transporter activity	28	1.05E-16	1.36E-14
GO:0015399	MF	primary active transmembrane transporter activity	28	1.34E-15	1.53E-13
GO:0019829	MF	ATPase-coupled cation transmembrane transporter activity	27	6.83E-23	1.89E-20
GO:0022853	MF	active ion transmembrane transporter activity	27	3.32E-21	5.59E-19
GO:0042625	MF	ATPase-coupled ion transmembrane transporter activity	27	3.32E-21	5.59E-19
GO:0015662	MF	P-type ion transporter activity	18	1.22E-14	1.24E-12
GO:0008171	MF	O-methyltransferase activity	12	3.45E-10	1.76E-08
