# Supplementary material for: Population genomics provides insights into the genetic diversity and adaptation of the Pieris rapae in China
Source: PLoS One. 2023 Nov 16;18(11):e0294521. doi: 10.1371/journal.pone.0294521 (PMC10653512; doi:10.1371/journal.pone.0294521)
Supplement: S10 Table — (PDF) [file pone.0294521.s014.pdf]

Table S10 Enriched KEGG pathway of selected genes in north population

ID	Kegg_pathway	GeneRatio	Pvalue	Corrected_P-value	Gene_number
ko04624	Toll and Imd signaling pathway	39/243	1.21E-29	2.72E-27	39
ko04722	Neurotrophin signaling pathway	19/243	1.80E-08	1.35E-06	19
ko04974	Protein digestion and absorption	24/243	2.98E-07	1.67E-05	24
ko00981	Insect hormone biosynthesis	9/243	4.33E-06	0.000194	9
ko04080	Neuroactive ligand-receptor interaction	22/243	5.72E-06	0.000213	22
ko00440	Phosphonate and phosphinate metabolism	5/243	1.65E-05	0.000491	5
ko04960	Aldosterone-regulated sodium reabsorption	8/243	1.75E-05	0.000491	8
ko04970	Salivary secretion	17/243	2.30E-05	0.000572	17
ko04925	Aldosterone synthesis and secretion	17/243	5.02E-05	0.001124	17
ko04110	Cell cycle	13/243	0.000577	0.010778	13
ko04022	cGMP-PKG signaling pathway	17/243	0.001676	0.025024	17
